# Quality of life in patients with inflammatory bowel disease: the role of positive psychological factors

**DOI:** 10.1080/21642850.2021.2007098

**Published:** 2021-11-26

**Authors:** Rafaela Matos, Leonor Lencastre, Vânia Rocha, Sandra Torres, Filipa Vieira, Maria Raquel Barbosa, Jorge Ascenção, Marina Prista Guerra

**Affiliations:** a Faculdade de Psicologia e de Ciências da Educação da Universidade do Porto, Porto, Portugal; b Centro de Psicologia da Universidade do Porto, Porto, Portugal; c Associação Portuguesa de Doença Inflamatória do Intestino, Porto, Portugal

**Keywords:** Inflammatory bowel diseases, meaning in life, body image, attachment, quality of life

## Abstract

**Objective:**

To identify differences in quality of life (QoL) of patients with inflammatory bowel disease (IBD) between diagnosis (Crohn’s Disease and Ulcerative Colitis), gender (male and female), treatment condition (with and without surgery), and attachment styles (secure, preoccupied, and disconnected); to examine associations between QoL, sociodemographic, clinical, and positive psychological variables; to determine whether sociodemographic, clinical, and positive psychological variables predict QoL.

**Method:**

The sample included 70 participants diagnosed with IBD (*Mage* = 43.37 years, *SD* = 12.81), of whom 71.4% were females and 67.1% had Crohn’s Disease. Positive psychological variables (meaning in life, positive body image, and attachment styles), sociodemographic (age, education, gender) and clinical variables (diagnosis, disease duration, surgery) were assessed as independent variables. QoL was the dependent variable, analyzed through four domains (physical, psychological, social, environment).

**Results:**

Participants with a secure attachment style reported higher QoL (physical, psychological, and social) than participants with a preoccupied attachment style. Strong positive correlations were found between positive psychological variables and QoL. Body appreciation was a significant predictor of three QoL domains (physical, psychological, and environment). Meaning in life made a unique contribution to the social QoL regression model, and it was also a significant predictor of psychological QoL. Body acceptance by others was a significant predictor of physical QoL, whereas disease duration and education predicted environment QoL. Attachment styles did not predict any QoL domain.

**Conclusion:**

The most significant predictors of QoL in patients with IBD were body appreciation and meaning in life. Body acceptance by others and body appreciation were the main predictors of physical QoL. Psychological interventions for patients who suffer from IBD should address body appreciation and meaning in life.

## Introduction

Inflammatory Bowel Disease (IBD) represents a group of gastrointestinal disorders that cause chronic and recurrent inflammation of the gastrointestinal tract (Knowles et al., 2018a). The main symptoms of IBD include abdominal pain and/or discomfort associated with diarrhea, changes in the shape of stools, rectal bleeding, and the presence of mucus in the stools (Salvioli & Bazzocchi, 2006). IBD encompasses two types of idiopathic intestinal diseases that share similar characteristics regarding motor and sensory physiology: Crohn’s Disease (CD) and Ulcerative Colitis (UC; Roda et al., 2020). The incidence of IBD is generally associated with a low quality of life (QoL), emotional distress, and high medical care costs (Gracie, Hamlin, & Ford, 2019; Parra et al., 2019). The morbidity inherent to the disease and the therapy itself, such as medication and surgeries, affect patients’ professional and school productivity, socialization, personal development, and family relationships. In addition, both diseases are associated with an increased risk of developing intestinal cancer and, thus, with consequent mortality (Knowles et al., 2018a).

Evidence suggests that altered gut-brain axis may affect the course and development of IBD, and may lead to psychological symptoms, such as chronic abdominal pain, anxiety and depression (Regueiro, Greer, & Szigethy, 2017). There are multiple factors associated with psychological symptoms among patients with IBD. For instance, anxiety may occur due to perceived stress, abdominal pain, and is associated with a low socioeconomic status. Depressive symptoms can be related with perceived stress, but also with invasive exams, such as endoscopy, and hospital admissions (Goodhand et al., 2012).

The prevalence of IBD in Portugal was 146 per 100,000 inhabitants, according to a study conducted between 2003 and 2007 (Azevedo et al., 2010). The authors of this study acknowledged the difficulty to conduct population studies and to update data on the prevalence of IBD due to its low incidence. However, there is evidence of an increased prevalence of IBD worldwide, and this growth seems to be associated with globalization and industrialization, as the highest prevalence rates of IBD are reported in North America and Europe (Gasparetto & Guariso, 2013).

QoL of patients diagnosed with IBD can be affected by multiple factors, such as emotional distress and high medical care costs (Drossman, Camilleri, Mayer, & Whitehead, 2002). Patients may also have concerns about a possible impairment of their daily functioning, even during remission phases (Keeton, Mikocka-Walus, & Andrews, 2015). In fact, IBD symptoms can cause significant physical, emotional, and social changes. Some patients identified the onset of emotional and QoL changes when they started experiencing symptoms (Souza, Barbosa, Espinosa, & Belasco, 2011). Studies showed that QoL is significantly lower among individuals (adults and children) diagnosed with IBD when compared to healthy controls (Knowles et al., 2018a). QoL, particularly the psychological domain, is even lower during the IBD active phase of symptoms (Knowles et al., 2018a, 2018b). As concluded in a recent systematic review, there is no consensus in the literature regarding differences in QoL between subtypes of IBD (Knowles et al., 2018b). In a later study, Amorim and Guerra (2018) did not find statistically significant differences in QoL of patients diagnosed with CD and UC.

The literature showed that patients diagnosed with chronic diseases who sought and experienced ML, reported higher levels of acceptance and well-being, lower levels of psychological distress, closer and more satisfying interpersonal relationships, and used effective coping strategies (Dezutter et al., 2013; Sherman & Simonton, 2012) Thus, ML can be positively related to patients’ psychological adjustment to severe medical conditions (Sherman & Simonton, 2012). Meaning in life (ML) is described by Guerra, Lencastre, Silva, and Teixeira (2017) as the perception of having goals in life and the mission to pursue and develop one’s own potentials. Viktor Frankl, a pioneer in ML research, believed that suffering could become an opportunity for personal growth, as a result of lessons learned from adverse situations (Sommerhalder, 2010).

Guerra et al. (2017) found a positive association between ML and QoL, and a negative association between ML and depression/anxiety among patients with colorectal cancer. Reis, Lencastre, Jonsson, and Guerra (2020) concluded that ML was a significant predictor of QoL of patients diagnosed with HIV/AIDS, particularly of the psychological and environment domains. ML and anxiety were also found to be significant predictors of psychological and environment QoL of patients diagnosed with multiple sclerosis (Pinto & Guerra, 2018). Global characteristics of ML were also found among patients diagnosed with IBD (Purc-Stephenson, Bowlby, & Qaqish, 2015), however, ML was not specifically assessed. ML will be studied for the first time in IBD due to its importance for adjustment to a variety of diseases, according to the literature review.

Body image has started to be studied in several chronic diseases, including in IBD (Moreira & Canavarro, 2010). A positive body image is defined as the ability to maintain positive opinions about one’s own body despite any particularity in physical appearance, to accept and respect one’s own body despite weight and imperfections, to adopt health behaviors, and to reject the unrealistic body images perpetuated by the media (Tylka & Wood-Barcalow, 2015). Patients diagnosed with IBD often express great concern about their body image and about the physical changes associated with the disease, such as weight loss (Keeton et al., 2015). A negative body image can cause avoidance of situations where exposure of the body or parts of the body is required, such as swimming or drawing blood for laboratory testing (Thomas, 2009). A negative body image is associated with a low physical and psychological QoL in patients with IBD (Trindade, Ferreira, & Pinto-Gouveia, 2017). Conversely, a positive body image is positively correlated with various well-being indices, such as self-esteem, life satisfaction (Swami, Tudorel, Goian, Barron, & Vintila, 2017), quality of life (Lemoine et al., 2018), and positive affect (Razmus & Razmus, 2017). However, given that the construct definition of positive body image is recent, the link between this variable and other psychological dimensions in patients with chronic diseases is not yet established.

Attachment styles have been studied in different clinical populations, as well as in patients diagnosed with IBD (McWilliams & Bailey, 2010). Attachment Theory (Bowlby, 1973) assumes that children internalize experiences with their caregivers in a way that the attachment style becomes a basis for how they will relate to others in the future. Attachment styles are considered more or less stable during the life cycle. Romantic partners and close friends can become the main attachment figures during adolescence and adulthood (Shaver & Mikulincer, 2010). When an adult’s attachment figure is responsive, sensitive and available towards proximity-seeking behaviors, the person experiences security and increases confidence in using proximity-seeking behaviors as an effective coping strategy (Shaver & Mikulincer, 2010)

The predominant attachment style among patients diagnosed with IBD is the fearful followed by the preoccupied style (Agostini et al., 2010; Ercolani et al., 2010). High levels of need for approval and concern about relationships were found among individuals diagnosed with CD. Some individuals perceived their parents as overprotective and their mothers as unresponsive during childhood. These findings suggest that subjects with fearful and preoccupied attachment styles have a negative view of their self, resulting in high levels of dependence and low self-esteem (Agostini et al., 2010). In addition, patients with UC reported higher levels of anxiety, need for approval, and avoidance of relationships compared to healthy controls. Concern about relationships was found to be a predictor of stress in this clinical population (Agostini, Spuri-Fornarini, Ercolani, & Campieri, 2016). These results corroborate that patients with IBD who report a negative physical and psychological QoL usually present a fearful or a preoccupied attachment style (Agostini et al., 2014). In fact, the preoccupied and fearful attachment styles have been associated with the emergence of psychosomatic illnesses and with the worsening of chronic symptoms.

Thus, positive attachment styles, ML, and a positive body image are associated with QoL of patients diagnosed with IBD (Ercolani et al., 2010; Purc-Stephenson et al., 2015; Trindade et al., 2017). In sum, despite the negative consequences of the disease, some patients were able to identify positive factors, such as personal growth, appreciation of life, perception of new paths to be taken, spiritual growth, and improvement of interpersonal relationships (Purc-Stephenson et al., 2015). Therefore, it is important to assess positive psychosocial factors that may facilitate the adjustment to the disease and improve patients’ QoL, such as meaning in life, positive body image, and secure attachment style (Drossman et al., 2002). This study aimed: (1) to identify differences in QoL of patients diagnosed with IBD regarding diagnosis (CD and UC), gender (male and female), treatment condition (with and without surgery), and attachment styles (secure, preoccupied, and disconnected); (2) to examine the associations between QoL, positive psychological variables (ML, positive body image, attachment style factors), and sociodemographic and clinical variables (age, education, disease duration); (3) to determine if sociodemographic, clinical, and positive psychological variables are predictors of QoL in patients diagnosed with IBD.

## Materials and methods

### Participants

Seventy-three participants were recruited from the Portuguese Association of Inflammatory Bowel Disease (APDI). Patients were included in the study if they were 18 years of age or older, were diagnosed with IBD, and did not suffer from any other chronic condition. Three participants did not meet the inclusion criteria, as they presented other comorbidities, such as obesity, obsessive-compulsive disorder, and psoriasis. The final sample was thus composed of 70 participants.

Table 1 shows that 71.4% of the participants were females and 67.1% were diagnosed with CD. The mean age was 43.37 (*SD *= 12.81), ranged from 18 to 73 years of age. Most participants were married (50%; *n* = 35). The mean years of schooling was 15.16 (*SD *= 3.51), with an education range of 7 (basic education) to 25 years (higher education).

**Table 1.** Descriptive statistics for sociodemographic and clinical variables.

	*n*	%	*M*	*SD*	*Range*
IBD diagnosis					
Crohn’s disease	47	67.1			
Ulcerative Colitis	21	30.0			
Undefined	2	2.9			
Age	70		43.37	12.81	18–73
Gender					
Female	50	71.4			
Male	20	28.6			
Marital status					
Single	27	38.6			
Married	35	50.0			
Divorced	8	11.4			
Education (years)	70		15.16	3.51	7–25
Disease duration (months)missing	68		163.78	128.15	2–500
Missing treatment	2				
Treatment					
Surgery	19	27.1			
No surgery	51	72.9			

Note: IBD: inflammatory bowel disease.

The mean of months elapsed between receiving the diagnosis and the data collection (disease duration) was approximately 164 (*SD *= 128.15; range 2–500 months). Thus, the sample included participants who were recently diagnosed, but also individuals who have lived with the diagnosis for 41 years. Most participants have not been submitted to surgery (72.9%; *n *= 51).

### Measures

#### Quality of life

The WHO Quality of Life Scale Abbreviated Version (WHOQOL-Bref; Vaz Serra et al., 2006; WHOQOL Group, 1998) was used to assess QoL, which includes 26 questions and four domains: (1) physical, (2) psychological, (3) social, and (4) environment. Participants rated items on a five-point Likert scale that ranged from one to five and measured intensity, frequency, evaluation, and capacity. WHOQOL-Bref domains scores vary between zero and 100 points, with higher scores indicating higher QoL. In the current study, the Cronbach’s alpha of domain 1 was .83, domain 2 was .83, domain 3 was .71, and domain 4 was .79.

#### Meaning in life

Meaning in Life Scale (Guerra et al., 2017) was used to assess ML and includes seven items (e.g. I am interest in life and I make plans). Participants rated items on a five-point Likert scale that ranged from one (strongly agree) to five (strongly disagree). The total score was obtained by summing all seven items (comprising reversed items). Higher values on this scale describe higher levels of ML. Reliability was adequate in the current study (*α* = .85).

#### Positive body image

Two scales were used to measure positive body image. The Body Appreciation Scale-2 (BAS-2) (Lemoine et al., 2018; Torres, Barbosa, Meneses, Tylka, & Vieira, 2018) was used to assess body appreciation, which includes ten items (e.g. I respect my body). Participants rated items on a five-point Likert scale that ranged from one (never) to five (always). Higher values on this scale describe higher levels of body appreciation. Reliability was adequate in the current study (*α* = .93). The Body Acceptance by Others Scale (BAOS; Barbosa et al., 2018; Meneses, Torres, Miller, & Barbosa, 2019) was used to assess participants’ perception of body acceptance by others, which includes 10 items. Participants rated items on a five-point Likert scale that ranged from one (never) to five (always). The total score was calculated as a mean of the 10 items. Higher values on this scale describe higher levels of perceived body acceptance. Reliability was adequate in the current study (*α* = .93).

#### Attachment styles

The Adult Attachment Scale was used to assess attachment styles (Canavarro, Dias, & Lima, 2006), which includes 18 items and 3 factors: Anxiety; Trust in others; and Comfort with proximity. Participants rated items on a five-point Likert scale that ranged from one (not at all characteristic of me) to five (extremely characteristic of me). Each factor total score was calculated as a mean of the items included in that factor (comprising a total of 7 reversed items). Individuals with mean scores above 3 in the Comfort and Trust factors and below 3 in the Anxiety factor were classified as ‘Secure’, those with mean scores above 3 in the Comfort and Trust factors and above 3 in the Anxiety factor were classified as ‘Preoccupied’, those with mean values below 3 in the Comfort and Trust factors and below 3 in the Anxiety factor were considered ‘Disconnected’, and those with mean values below 3 in the Comfort and Trust factors and above 3 in the Anxiety factor were considered ‘Fearful’. In the current study, the Cronbach’s alpha of factor 1 was .89, factor 2 was .65, and factor 3 was .54.

### Procedure

The current study is part of a larger project entitled ‘Body acceptance in disease: Study of positive body image in different clinical conditions’, which was approved by the ethic committee at the Faculty of Psychology and Educational Sciences (FPCEUP) (Ref. 2018/12-6; date of approval: 18th December 2019). Data was collected in collaboration with the Portuguese Association of Inflammatory Bowel Disease (APDI) between January and April 2020. Invitations to complete the survey were sent by APDI. The members received an email containing a brief description of the study and a link to a secure online survey. Participants were informed, online, about the ethical principles of the study (voluntary participation, protecting the confidentiality of data, the right to withdraw from the research at any time, and principal investigator contact). After providing digital informed consent, participants were asked to complete the instruments described above.

### Data analysis

Data were analyzed using the Statistical Package for the Social Sciences, 26.0 version for Windows (IBM SPSS *Statistics* 26).

Descriptive analysis was performed to characterize the sample and the variables under study. The assumption of normality was met with all variables. To test this assumption, we used the Kolmogorov–Smirnov test (KS; with Lilliefors Significance Correction) and the following criteria: absolute skewness (Sk) and kurtosis (K) values lower than 3.0 and 8.0, respectively (Kline, 2005). No missing data were found. Cronbach’s alpha was performed to assess the instruments internal consistency. Independent samples t-tests were conducted to compare the QoL scores in independent variables with two levels (diagnosis, gender, and treatment). Cohen (1988) statistic was used as a measure of effect size: effect sizes of .2 were considered small, of .5 were considered medium, and of .8 were considered large. The one-way between-group analysis of variance (ANOVA) was conducted to compare QoL scores in independent variables with more than two levels (attachment styles). Eta-squared (*η*^2^) was interpreted according to the commonly used Cohen’s (1988) guidelines: .01 = small effect, .06 = moderate effect, .14 = large effect. Pearson’s product-moment correlations were also performed to examine the strength of the association between the variables included in the study (QoL, sociodemographic and clinical variables: age, education, disease duration; positive psychological variables: ML, positive body image, and attachment styles), and were interpreted according to the following guidelines: *r* ≤ .10 = low correlations, *r* ≤ .30 = moderate correlations, *r* ≥ .50 = strong correlations (Cohen, 1988). Multiple linear regression analyses with the enter method were performed to identify which independent variables significantly predicted QoL domains, and their contribution to the model. Each independent variable was selected due to its theoretical relevance, to the significance levels found in ANOVA and in the univariate and bivariate analyses (independent variables that were strongly associated with QoL were included into the multiple regression models), and considering the rule of thumb for social science research of at least 15 subjects per predictor (Stevens, 1996 cited in Pallant, 2013). A significance level of .05 was considered, with a 95% confidence interval.

## Results

Descriptive statistics (percentages, means, standard-deviations, and range) for positive psychological variables are displayed in Table 2.

**Table 2.** Descriptive statistics for positive psychological variables under study.

	*N*	Minimum	Maximum	Mean	*SD*
Body acceptance	70	1.40	5.00	3.73	0.72
Body appreciation	70	1.60	5.00	3.79	0.84
Meaning in life	70	16.00	35.00	27.23	4.65
Anxiety (AF)	70	1.17	4.83	2.37	0.79
Comfort (AF)	70	1.83	4.50	3.43	0.55
Trust (AF)	70	1.67	4.00	3.11	0.53
Overall QoL	70	12.50	100.00	64.82	17.33
Physical QoL	70	25.00	96.43	65.92	16.81
Psychological QoL	70	20.83	95.83	71.37	15.41
Social QoL	70	00.00	100.00	63.81	19.03
Environment QoL	70	28.13	100.00	69.78	13.16

Note*:* AF: attachment factor; QoL: quality of life.

Table 3 shows the results regarding the comparison between different diagnosis (CD and UC) on each domain of QoL (physical, psychological, social and environment). The results showed that there were no significant differences between participants diagnosed with CD and UC regarding QoL scores. There were two subjects with undefined diagnosis who were not included in the analysis.

**Table 3.** Comparison of participants diagnosed with Crohn’s Disease and participants diagnosed with Ulcerative Colitis on QoL scores.

QoL	Crohn’s disease (*n *= 47)		Ulcerative Colitis (*n *= 21)					
	*M*	*SD*	*M*	*SD*	*t*	*df*	*p*	Cohen’s *d*
Physical	67.86	16.28	62.42	18.30	−1.23	66	.23	−.32
Psychological	72.52	15.36	68.25	15.78	−1.05	66	.30	−.28
Social	65.78	18.33	61.51	18.72	-.88	66	.38	−.23
Environment	71.34	12.69	66.22	12.91	−1.53	66	.13	−.40

Note: QoL: quality of life.

Table 4 shows the results regarding the comparison between gender (female and male) on each domain of QoL. No significant differences were found between female and male participants in the four domains of QoL.

**Table 4.** Comparison of female and male participants on QoL scores.

QoL	Female (*n *= 48)		Male (*n *= 20)					
	*M*	*SD*	*M*	*SD*	*t*	df	*p*	Cohen’s *d*
Physical	67.43	15.70	62.14	19.23	1.19	68	.24	.32
Psychological	70.58	15.86	73.33	14.40	−.67	68	.50	−.18
Social	62.67	18.38	62.67	20.77	−.79	68	.43	−.21
Environment	69.75	14.36	69.84	9.84	−.03	68	.98	−.01

Note: QoL: quality of life.

Table 5 shows the results regarding the comparison between different treatment conditions (with and without surgery) on each domain of QoL. No significant differences were found between individuals who underwent a surgery and participants who were not submitted to surgery regarding QoL scores.

**Table 5.** Comparison of participants submitted to surgery and participants who were not submitted to surgery on QoL scores.

QoL		No surgery (*n *= 51)		Surgery (*n *= 19)				
	*M*	*SD*	*M*	*SD*	*T*	*df*	*p*	Cohen’s *d*
Physical	65.34	17.66	67.48	14.62	−.47	68	.64	−.13
Psychological	69.93	15.71	75.22	14.26	−1.28	68	.20	−.35
Social	63.07	19.60	65.80	17.76	−.53	68	.60	−.14
Environment	69.55	13.85	70.39	11.43	−.24	68	.81	−.06

Note: QoL: quality of life.

The most frequent attachment style was secure (*n* = 43), followed by preoccupied (*n* = 13), disconnected (*n* = 11) and fearful (*n* = 3). Thus, subjects who presented a fearful attachment style were excluded from this analysis due to the small subsample size. Individuals with a secure attachment style reported a significantly higher physical (*F* (2.64) = 6.03, *p* = .004, η^2^ = .16), psychological (*F* (2.64) = 4.82, *p* = .011, η^2^ = .13), social QoL (*F* (2.54) = 6.57, *p* = .003, η^2^ = .17) than participants with a preoccupied attachment style, but there were no differences for the environment QoL (*F* (2,64) = 1.35, *p* = .226, η^2 ^= .41). Data not demonstrated.

Table 6 shows the results regarding the correlations between QoL domains and the independent variables under study (ML, body appreciation, body acceptance, factors regarding attachment styles (anxiety, comfort with proximity, and trust in others), age, education, and disease duration).

**Table 6.** Correlations between independent variables and the QoL domains.

	Physical QoL	Psychological QoL	Social QoL	Environment QoL
Meaning in life	.40**	.69**	.45**	.45**
Body appreciation	.61**	.68**	.38**	.60**
Body acceptance	.53**	.22	.35**	.43**
Anxiety (AF)	−.31**	−.45**	−.37**	−.19
Comfort (AF)	.29*	.36**	.29*	.27*
Trust (AF)	.44**	.38**	.34**	.34**
Age	.06	.17	−.02	.93
Education	.17	.28*	.20	.32**
Disease duration	.18	.13	−.02	.26*

Note: QoL: quality of life; AF: attachment factor; *p < .05; **p < .01.

All positive psychological variables were significantly correlated with QoL domains, with exception of psychological QoL that was not significantly correlated with body acceptance and of environment QoL domain that was not significantly correlated with anxiety attachment factor. Regarding sociodemographic and clinical variables, psychological (*r* = .28; *p* = .018) and environment QoL domains (*r* = .32; *p* <.01) were positively correlated with education. The environment domain was also found to be positively correlated with the disease duration (*r* = .26; *p* = .035).

Table 7 shows that body appreciation (*β* = .39, *t* (65) = 3.08, *p *< .01) and body acceptance (*β* = .26, *t* (65) = 2,41, *p *= .02) made a statistically significant contribution to the physical QoL regression model. The model was significant and explained 43.1% of the variance in physical QoL (F (65,4) = 14.042, *p* <.001). Variance Inflation Factor (VIF) values were analyzed, and it was concluded that the model did not exhibit multicollinearity.

**Table 7.** Multiple regression models for QoL.

Regression model	*β*	*t*	*p*	Adjusted *R^2^*	*df*	F	*p*
Physical QoL				.431	65	14.04	.00
Body appreciation	.39	3.08	.00				
Body acceptance	.26	2.41	.02				
Meaning in life	.02	.17	.86				
Trust (AF)	.18	1.81	.08				
Psychological QoL				.578	65	24.62	.00
Body appreciation	.36	3.48	.00				
Meaning in life	.43	4.41	.00				
Trust (AF)	.04	.39	.70				
Anxiety (AF)	−.10	−.97	.34				
Social QoL				.232	65	6.20	.00
Body appreciation	.01	.09	.93				
Body acceptance	.18	1.47	.15				
Anxiety (AF)	−.19	−1.60	.11				
Meaning in life	.30	2.31	.02				
Environment QoL (model 1)				.368	65	11.06	.00
Body appreciation	.41	3.12	.00				
Body acceptance	.13	1.18	.24				
Trust (AF)	.08	.74	.46				
Meaning in Life	.15	1.27	.21				
Environment QoL (model 2)				.437	67	18.30	.00
Body appreciation	.56	6.06	.00				
Education	.24	2.58	.01				
Disease duration	.19	2.02	.05				

Note: QoL: quality of life; AF: attachment factor.

Body appreciation (*β* = .36, *t* (65) = 3,48, *p *< .01) and ML (*β* = .43, *t* (65) = 4,41, *p *< .01) made a statistically significant contribution to the psychological QoL regression model. The model was significant and explained 57.8% of the variance in psychological QoL (F (65,4) = 24.622, *p *< .001). VIF values were analyzed, and it was concluded that the model did not exhibit multicollinearity.

The four variables that showed the highest correlation with social QoL (body appreciation, body acceptance by others, Anxiety factor of attachment styles, and ML) were included in this model. ML made a statistically significant contribution to the social QoL regression model (*β* = .30, *t* (65) = 2.31, *p *= .02). The model was significant and explained 23.2% of the variance in social QoL (F (65,4) = 6.198, *p* < .001). VIF values were analyzed, and it was concluded that the model did not exhibit multicollinearity.

Body appreciation, body acceptance by others, Trust in others, and ML were initially included into the environment QoL regression model, as they were the variables that showed the highest correlation with environment QoL. The results showed the model was significant and explained 36.8% of the variance in environment QoL (F (65,4) = 11.055, *p* < .001), but body appreciation was the only variable that made a unique contribution to the model (*β* = .41, *t* (65) = 3.12, *p *< .01). Therefore, we decided to improve the environment QoL regression model, by including the most relevant sociodemographic and clinical variables for this domain according to the literature. The second model explained a higher variance (43.7%) in environment QoL (F (67,3) = 18.304, *p* < .001). Body appreciation (*β* = .56, *t* (67) = 6,06, *p *< .01), education (*β* = .24, *t* (67) = 2.58, *p *< .01), and disease duration (*β* = 19, *t* (67) = 2.02, *p *= .05) made a statistically significant contribution to the environment QoL regression model. The model VIF values were analyzed, and it was concluded that the model exhibited no multicollinearity.

## Discussion

The present study aimed to identify differences in QoL according to patients’ diagnosis, gender, treatment condition, and attachment styles. No significant differences were found between CD and UC on QoL. This result is in line with the studies conducted by Amorim and Guerra (2018) and by Ribeiro (2014). On the other hand, other studies have found differences in QoL between patients with CD and UC in the active phase of the disease (Gavrilescu et al., 2015; Gil & Fernandes, 2019; Haapamäki, Turunen, Roine, Färkkilä, & Arkkila, 2009; Knowles et al., 2018b). These differences may be explained by the active or remission phases of the disease, but this information was not collected in the current study.

Regarding gender, some studies have reported a lower QoL in females diagnosed with IBD than in males (Haapamäki et al., 2009; Moradkhani, Beckman, & Tabibian, 2013), suggesting that psychological factors could play a greater role in females. According to Moradkhani et al. (2013), females are more likely to report concerns related to attractiveness and body image and rate their symptoms as being more severe. However, there is no consensus in the literature, as no differences were found in QoL between females and males in other studies (Amorim & Guerra, 2018; Gavrilescu et al., 2015) which also corroborates the results found in the current study.

No differences were found in QoL between participants submitted to surgery and participants not submitted to surgery. The purpose of surgical interventions is to increase QoL by controlling symptoms and decreasing pain. In fact, previous studies supported a significant improvement in QoL after surgical intervention among patients with IBD. This improvement was more pronounced in the first year after surgery in patients with CD due to the low incidence of postoperative disease recurrence reported during this period (Bączyk, Formanowicz, Gmerek, & Krokowicz, 2017; Thirlby, Land, Fenster, & Lonborg, 1998). However, fear about postoperative complications and adaptation, as well as concerns about decreased attractiveness and self-esteem, may arise during the decision to undergo surgery (Allison, Lindsay, Gould, & Kelly, 2013). Therefore, health professionals should help patients to use effective coping strategies to deal with these issues in order to benefit from the possible postoperative QoL improvement.

Lastly, the most frequent attachment style found in the current study was the secure style. This finding is not in line with most of the studies conducted with patients diagnosed with chronic diseases and with IBD, in which the most common attachment styles found were preoccupied and fearful styles (Agostini et al., 2010; Agostini et al., 2014; Agostini, Scaioli, Belluzzi, & Campieri, 2019; Ercolani et al., 2010; Matos, Barbosa, & Costa, 2001; McWilliams & Bailey, 2010). The securely attached participants may have been attracted to being part of a support organization (APDI), where other resources (such as psychological counseling and peer support) were available and may have facilitated adjustment to the disease. The results of this study showed significant differences between the secure and preoccupied attachment styles on the physical, psychological and social domains of QoL. Patients with a secure attachment style reported a higher QoL. These results are supported by Agostini et al. (2014) who found that the preoccupied attachment style among patients with IBD was associated with a lower physical and psychological QoL.

Regarding the associations between QoL and positive psychological variables, positive correlations between ML and all QoL domains were found. Therefore, ML can play an important role in enhancing patients’ adjustment to the disease and, consequently, in improving perceived QoL, as observed in other studies (Guerra et al., 2017; Reis et al., 2020; Sherman & Simonton, 2012). Body appreciation was positively correlated with all QoL domains, while perceived body acceptance by others was positively correlated with physical, social and environment QoL. These results are in line with the study of Trindade et al. (2017) that found an association between a less positive body image and lower scores on physical and psychological QoL domains in a Portuguese sample diagnosed with IBD. Trindade, Ferreira, Duarte, and Pinto-Gouveia (2019) reported correlations between IBD symptomatology and a negative body image, which reinforce the impact of body image on patients’ QoL. As in other clinical conditions, such as cancer disease or spinal cord injury (Bailey, Gammage, van Ingen, & Ditor, 2015; Moreira & Canavarro, 2010), these results suggest that acceptance of body changes, and valuing it beyond beauty ideals, are essential factors for a new post-disease identity, a better quality and meaning of life. Like Grogan, Mechan, Persson, Finlay, and Hall (2019) concluded, patients were able to develop a positive body image after suffering from an adverse clinical condition, as female participants with IBD and submitted to surgery started accepting and creating a new body identity after realizing their body was able to resist a severe disease.

Another goal of this study was to determine if positive psychological variables, as well as sociodemographic and clinical variables, were predictors of QoL in patients diagnosed with IBD. The regression models revealed that body appreciation and body acceptance by others were significant predictors of physical QoL, suggesting that this domain addresses mobility, daily living activities, and ability to work. Body appreciation and ML were found to be significant predictors of psychological QoL. The positive relationship with the body, the adoption of behaviors that promote well-being and personal growth tend to be associated with better health rates and adaptation to the disease, which, in turn, promote a better QoL (Ribeiro, 2014). Social QoL includes sexual activity, personal relationships, and social support, which may explain why ML was found to be significant predictor of this domain. The environment domain of QoL encompasses leisure opportunities, economic resources, opportunities to acquire new information and to develop new skills, which may explain why education is a significant predictor of this domain. Casellas, López-Vivancos, Casado, and Malagelada (2002) found that patients diagnosed with IBD with a higher level of education, reported higher levels of QoL. The disease duration was also a predictor of QoL environment domain. Evidence shows that patients who live with the diagnosis the longest, report a higher perceived QoL, whereas patients who were recently diagnosed, were more likely to experience anxiety and depressive symptoms (Casellas et al., 2002; Jäghult, Saboonchi, Johansson, Wredling, & Kapraali, 2011; van den Brink et al., 2018). Body appreciation was also found to be a significant predictor of this domain, as it reinforces that subjects who have more respect and a more positive attitude towards their body, may also address quality and availability of health care in a more positive manner (environmental QoL).

The current findings should be interpreted in light of both strengths and limitations. One of the strengths of the current study is that it focuses on positive psychological variables, such ML and a positive body image, which are understudied in individuals with IBD. Thus, it provides a constructive approach of QoL among individuals who suffer from a chronic disease. In addition, positive body image was assessed by two instruments focused on body appreciation and perceived body acceptance by others, which differs from most studies that focus on the negative aspects of body image perception in clinical samples. The high prevalence of the secure attachment style in this study might act as a protective factor for QoL in Portuguese individuals diagnosed with IBD. This finding can create opportunities to deepen the study of this phenomenon in the future. Barbosa, Matos, and Costa (2011) found that positive attachment to parents and to romantic partners was a significant predictor of a positive body image in a sample of young Portuguese adults. These results suggest that a positive body image can also be influenced by attachment styles. In future studies, it would be useful to determine if attachment styles are predictors of a positive body image in patients diagnosed with IBD or with other chronic health conditions.

This study has also some limitations. Control group was not used to verify whether the values diverged significantly when compared to healthy controls. The limitations of the sample (namely sample size, the sampling method, and the sociodemographic characteristics of the sample) may limit the generalization of the results to the Portuguese population diagnosed with IBD. Thus, it would be important to collect a larger sample with more heterogeneity regarding gender in order to achieve more reliable conclusions. It would also be important to collect data about the stage of the disease (active or remission) to better understand if this variable contributes to the improvement of QoL (Tylka & Wood-Barcalow, 2015). Covid-19 pandemic started during the data collection period. It would had been useful to identify differences between patients who answered the protocol before and after this moment, as literature reveals alarming social impact and declining follow-up care for IBD patients during the covid-19 outbreak (Feitosa et al., 2021).

## Conclusions

This study added a positive perspective about the factors that influence QoL of individuals diagnosed with IBD. It also enhances the need to promote, in clinical practice, a positive body image of patients, to help them seek for ML, and build positive attachment styles, in order to improve QoL. The findings revealed that all independent variables included in this study were significant predictors of at least one of the four domains of QoL, which reinforces the protective factors complexity in patients with a chronic disease. This study can contribute to the advancement of scientific research in the field of positive psychology and to the development of clinical practices that help improving QoL in patients with IBD.
